# Trismus Pseudocamptodactyly Syndrome: A Sporadic Cause of Trismus

**DOI:** 10.1155/2013/187571

**Published:** 2013-09-12

**Authors:** Prathima Sreenivasan, Faizal C. Peedikayil, Sumal V. Raj, Manasa Anand Meundi

**Affiliations:** ^1^Department of Oral Medicine and Radiology, Kannur Dental College, Anjarakkandy, Kannur, Kerala 670612, India; ^2^Department of Pedodontics, Kannur Dental College, Anjarakkandy, Kannur, Kerala 670612, India; ^3^Department of Oral Medicine and Diagnostic Radiology, Sri Sankara Dental College, Akathumuri, Varkala, Kerala 695318, India; ^4^Department of Oral Medicine and Radiology, Dayananda Sagar College of Dental Sciences, Shavige Malleshwara Hills, Kumaraswamy Layout, Bangalore 560078, India

## Abstract

Trismus pseudocamptodactyly syndrome is a very rare autosomal dominant inherited disorder characterized by the inability to completely open the mouth (trismus) and the presence of abnormally short tendon units causing the fingers to curve (camptodactyly). Early diagnosis and management of this condition is important to prevent facial deformities in the patient. Reporting such a case is important as case reports are one of the sources of data for calculating the prevalence of rare diseases. Here, we report a case of trismus pseudocamptodactyly syndrome in an eight-year-old boy with a brief review of the literature.

## 1. Introduction 

Trismus pseudocamptodactyly syndrome (TPS) is a rare autosomal dominant disorder with sporadic incidence [1]. This condition was first reported by Hecht and Beale in 1969 [2] and was named the trismus pseudocamptodactyly syndrome (TPS). It is a type of Distal Arthrogryposis (arthro means joint; grypos means curved). They are a group of autosomal dominant disorders that mainly involve the distal parts of the limbs and also affect the temporomandibular joint leading to congenital deformities. They are characterized by congenital contracture of two or more different areas without a primary neurological or muscular disease [1, 3]. Ten different arthrogryposes have been described till date [1, 3, 4] (Table 1). Diagnostic criteria have been formulated for the diagnosis of each type of distal arthrogryposis. For the upper limb, major diagnostic criteria include camptodactyly or pseudocamptodactyly (limited passive proximal interphalangeal joint extension with hyperextension of the wrist), hypoplastic and/or absent flexion creases, overriding fingers, and ulnar deviation at the wrist [1, 4]. For the lower limb, major diagnostic criteria are talipes equinovarus, calcaneovalgus deformities, vertical talus, and/or metatarsus varus. To be considered affected, an individual must exhibit two or more of these major criteria. When a first-degree family member (a parent or a sibling) meets these diagnostic criteria, a person with at least one major diagnostic criterion is considered affected [1, 3, 4]. In the revised and extended classification scheme of distal arthrogryposis, Bamshad et al. classified TPS as distal arthrogryposis type 7 (DA7) [4]. The major diagnostic criteria are trismus and pseudocamptodactyly [3, 4]. The features of distal arthrogryposis type 7 (DA7) are limited excursion of the mandible, shortened muscle tendon units of the hands (consequently, flexion deformity of the fingers that occurs with wrist extension, camptodactyly), and foot deformities related to shortened muscle legs thereby preventing normal growth and development [1, 2, 4]. 

## 2. Case Report 

An eight-year-old boy was reported with a complaint of difficulty in opening the mouth. History revealed that the patient noticed gradual reduction in mouth opening for the last one year. Mouth opening had decreased rapidly in the last one month. Family history revealed that his parents had a consanguineous marriage. Mother had pregnancy induced hypertension in the antenatal period due to which a caesarian section was performed. Child was delivered preterm at seven months of gestation, and birth weight was 1.4 kg. Contractures of fingers, knees, and toes were noted at birth. Delay in achieving milestones both motor and speech was obvious. Patchy hair loss and rampant caries were noticed for the last two years. The boy underwent surgical release of the knee contractures at the age of four after which walking was achieved. The contractures in the leg have improved since then. History revealed that his sibling was also a preterm child and died at the fourth day after delivery. 

**Figure 1:**
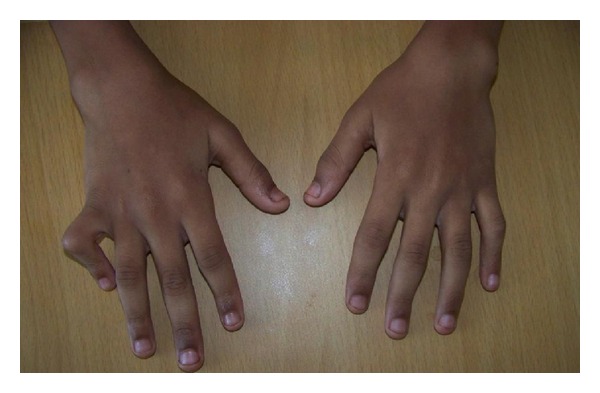
Photograph of hand showing camptodactyly-curved little finger.

**Figure 2:**
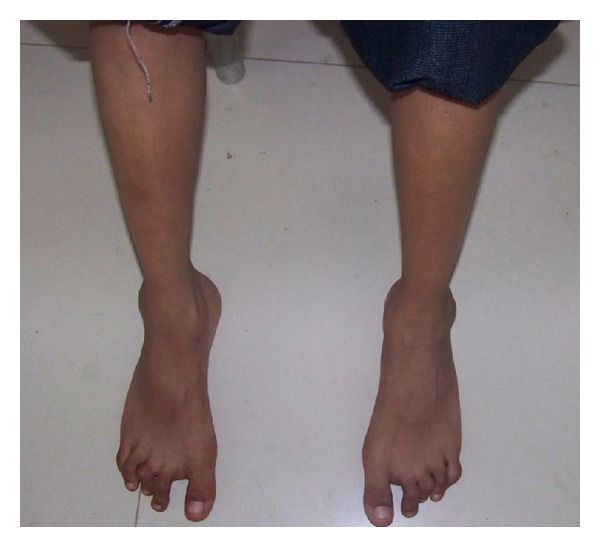
Photograph of feet showing contracture of all the toes and valgus deformity of the feet.

 On examination, the patient had normal height and weight. On central nervous system (CNS) examination Memory, intelligence and affect were normal. Hypertonia of both lower limbs with brisk reflexes was noted. Upper limbs showed mild rigidity with normal reflexes. Camptodactyly was present (Figure 1). Contractures of all the toes with valgus deformity were also noted (Figure 2). Hypopigmented macules were noted on the back, buttocks, and both arms. Alopecia areata was also noted. 

**Figure 3:**
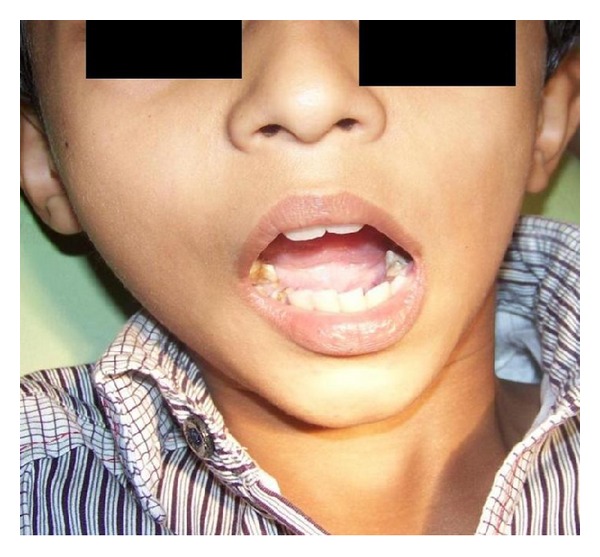
Decreased mouth opening with deviation towards left side.

 Malar hypoplasia and retrognathic mandible were present. Mouth opening was 20 mm. Deflection to the left during mouth opening was noted (Figure 3). On intraoral examination, caries involving all the deciduous posterior teeth were noted. 

### 2.1. Radiographic Investigations

**Figure 4:**
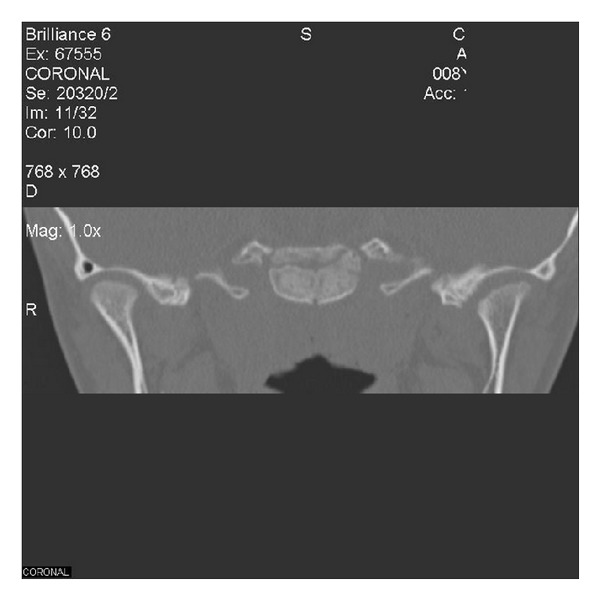
Coronal sectional CT showing flattening of the left condyle.

Altered shape and flattening of the left condyle was notable in the coronal sectional CT (Figure 4). 

### 2.2. Blood and Biochemistry Parameters Were within Normal Limits

The diagnosis of trismus pseudocamptodactyly syndrome/distal arthrogryposis type 7 was made based on the history of presence of congenital contractures, clinical findings of trismus, camptodactyly, valgus deformities of the feet, and correlating radiographic features of hand and the left condyle. 

 His parents were counseled regarding the temperomandibular joint (TMJ) problem and advised to undergo surgery at the earliest to remove the contracture, relieve the trismus, and prevent mandibular growth retardation. 

## 3. Discussion

Trismus pseudocamptodactyly syndrome is a rare autosomal disorder with sporadic incidence. The disease has variable expressivity, and the severities of clinical features vary widely. Other than trismus and camptodactyly, additionally reported features include shortened hamstring muscles and short stature. No single feature, including either trismus or pseudocamptodactyly, is present in all affected individuals [3, 4]. It does not present uniformly in all patients making it difficult to diagnose. Fibrosis of the muscles of the TMJ, thickening and shortening of periarticular capsular and ligamentous tissues, coronoid hyperplasia [5–7], and condylar deformities with reduced joint space [8–11] have been cited as reasons for contracture in the muscles. Fibrosis of the muscles of TMJ and condylar deformities could be the reason for trismus in our patient.

 Genetic studies have proven that it is caused by a single missense mutation in MYH8 that is predicted to cause an arginine-to-glutamine substitution in perinatal myosin. It is inherited in an autosomal dominant pattern [12]. The diagnosis is essentially clinical, and genetic analysis can be used only as an adjunct [4].

 The sustained contracture in the masticatory muscles leads to trismus, difficulties in speech, mastication, and mandibular growth retardation. Early diagnosis and detection of TMJ related changes are important in the management of the condition. Though the condition was diagnosed early, the TMJ findings were not detected initially in our patient. The contracture of the masticatory muscles resulted in trismus and functional difficulties which could have been avoided if he had been assessed for TMJ involvement in the initial stages.

 Surgical management is the treatment of choice [5–11]. Our patient was also advised surgical release of contracture in the masseter muscle and correction of condylar deformities to improve mouth opening and prevent further mandibular growth retardation. 

## 4. Conclusion

 Only a few cases of this condition have been reported in the literature till now. Improved knowledge will allow increased recognition and diagnosis, help in establishing prognosis, provide family counseling, and facilitate treatment. Early detection and appropriate intervention can prevent serious growth retardation and facial deformities. All children diagnosed with distal arthrogryposis should undergo periodic oral and maxillofacial assessment to rule out TMJ involvement. DA7/TPS should be included as a differential diagnosis for long standing trismus in children and adults. 

## Figures and Tables

**Table 1:** Classification of distal arthrogryposes.

Syndrome	New label	OMIM number
Distal arthrogryposis type 1	DA1	108120
Distal arthrogryposis type 2A Freeman Sheldon syndrome	DA2A	193700
Distal arthrogryposis type 2B Sheldon Hall syndrome	DA2B	601680
Distal arthrogryposis type 3 Gordon syndrome	DA3	114300
Distal arthrogryposis type 4 Scoliosis	DA4	609128
Distal arthrogryposis type 5 Ophthalmoplegia, ptosis	DA5	108145
Distal arthrogryposis type 6 Sensorineuronal hearing loss	DA6	108200
Distal arthrogryposis type 7 Trismus pseudocamptodactyly	DA7	158300
Distal arthrogryposis type 8 Autosomal dominant pterygium syndrome	DA8	178110
Distal arthrogryposis type 9 Congenital contractural arachnodactyly	DA9	121050
Distal Arthrogryposis type 10 Congenital Planar contractures	DA10	187370
